# Evaluation of an Intervention to Address Smoking and Food Insecurity at Preoperative Surgical Clinic Appointments

**DOI:** 10.1001/jamanetworkopen.2022.38677

**Published:** 2022-10-27

**Authors:** Alisha Lussiez, Alexander Hallway, Maxine Lui, Jose Perez-Escolano, Deena Sukhon, William Palazzolo, Hatim Elhady, Michael Englesbe, Ryan Howard

**Affiliations:** 1 Department of Surgery, Michigan Medicine, Ann Arbor; 2 Michigan Opioid Prescribing and Engagement Network, Ann Arbor; 3 Center for Healthcare Engineering and Patient Safety, University of Michigan, Ann Arbor

## Abstract

**Importance:**

The health of the US population is decreasing, and novel strategies are needed to reverse the trajectory. The surgical episode may be an opportune time to screen for poor health behaviors and social needs, yet little is known about the feasibility or acceptability of such efforts.

**Objective:**

To assess the feasibility and acceptability of a pilot program that engages patients in health behavior change and addresses unmet social needs at the time of surgery.

**Design, Setting, and, Participants:**

This quality improvement study was conducted among 10 338 patients seen in a preoperative clinic for elective surgery between February 8 and August 31, 2021. Patients who received a referral for institutional resources were contacted via telephone to complete follow-up surveys 30 to 90 days after surgery and between July 1, 2021, and March 31, 2022.

**Interventions:**

Implementation of a tool to screen patients for smoking and food insecurity in a preoperative clinic. Those who screened positive were offered referrals for institutional resources. Telephone surveys were conducted with patients who accepted referrals to understand attitudes toward addressing health behaviors and social needs.

**Main Outcomes and Measures:**

Screening and referral rates, patient-perceived acceptability of addressing health behaviors and social needs at the time of surgery, smoking cessation rates, and resolution of food insecurity.

**Results:**

A total of 10 338 patients (6052 women [58.5%]; mean [SD] age, 56.5 [17.9] years) were evaluated in the preoperative clinic. Of the 10 338 patients, 7825 (75.7%) were successfully screened. Of the 641 identified smokers, 152 (23.7%) accepted a referral for smoking cessation counseling. Of the 181 identified patients with food insecurity, 121 (66.9%) accepted a referral for nonmedical needs assistance. On follow-up surveys, 64 of 78 smokers (82.1%) agreed that the preoperative appointment was an appropriate time to discuss smoking cessation, and 34 of 78 smokers (43.6%) reported quitting smoking. Similarly, 69 of 84 patients with food insecurity (82.1%) agreed it was a good or very good idea for health systems to address nonmedical needs at the time of surgery, and 27 patients (32.1%) reported no longer being insecure about food since their preoperative visit.

**Conclusions and Relevance:**

This study suggests that it is feasible to address patients’ foundational health at the time of surgery. Most patients agreed that these interventions were appropriate during the perioperative period. These results support using the surgical episode as an opportunity to address foundational health.

## Key Points

**Question:**

Can patients’ foundational health be addressed at the time surgery?

**Findings:**

In this quality improvement study of 10 338 patients seen in a preoperative clinic for elective surgery, more than 75% of patients used the screening and referral tool during the preoperative visit to address active smoking and food insecurity. During follow-up, 82% of smokers and 82% of patients with food insecurity agreed it was a good idea for health systems to address their respective smoking or food-related needs around the time of surgery.

**Meaning:**

This study suggests that it is feasible and acceptable to engage patients in health behavior change and to address unmet social needs at the time of surgery.

## Teaser

This quality improvement study assesses the feasibility and acceptability of a pilot program that engages patients in foundational health behavior change and addresses unmet social needs at the time of surgery.

## Introduction

Compared with other developed countries, the United States has the highest spending on health care but the lowest performance on measures of population health.^1^ For example, the US has the shortest life expectancy, greatest burden of chronic disease, and highest rate of preventable death compared with other high-income countries.^2^ These outcomes are associated with health behaviors and social determinants of health, which account for 70% of all health outcomes.^3,4^ Poor health behaviors, such as smoking, and unmet social needs, such as food insecurity, are leading risk factors for premature death in the US.^5,6,7^ Although these issues have traditionally been addressed in the setting of primary care, the growing shortage of primary care physicians has further strained the response to these challenges.^8,9^ Innovative strategies are needed to mitigate these fundamental risks and improve the health of the US population.

Although receipt of specialty care, such as surgery, may be an opportune time to intervene on these issues, this approach remains largely unexplored, to our knowledge. Surgery is a transformative life event.^10,11^ The experience of undergoing surgery can serve as a teachable moment, during which patients are particularly receptive and motivated to achieve sustained health behavior change.^12,13^ For example, prior work has found that the tobacco quit rate among patients undergoing surgery is several times higher than the national average, even for operations unrelated to smoking.^14,15^ Despite the potential benefits associated with capitalizing on the teachable moment of surgery, there are barriers to this approach at the hospital, clinician, and patient level. At the hospital level, population health is underemphasized within specialty care.^16^ At the clinician level, time constraints and lack of training are cited as limitations to engaging patients in these domains.^17,18^ Patients themselves may be reluctant to discuss nonclinical needs around the time of a stressful operation. Little is known about the feasibility and patient perceptions of efforts to address population health needs at the time of surgery.

Therefore, we developed a program to study the feasibility and patient perceptions of preoperative engagement in health behaviors and unmet social needs. Specifically, we targeted smoking and food insecurity given their relatively high prevalence and well-established association with poor long-term health outcomes.^19^ After implementation of a screening and referral program to identify and connect patients with resources in these areas, patients were surveyed to understand their experience of this program. We hypothesized that patients would favorably view the perioperative period as an appropriate time to address and improve foundational health.

## Methods

### Pilot Design and Setting

**Figure 1.**
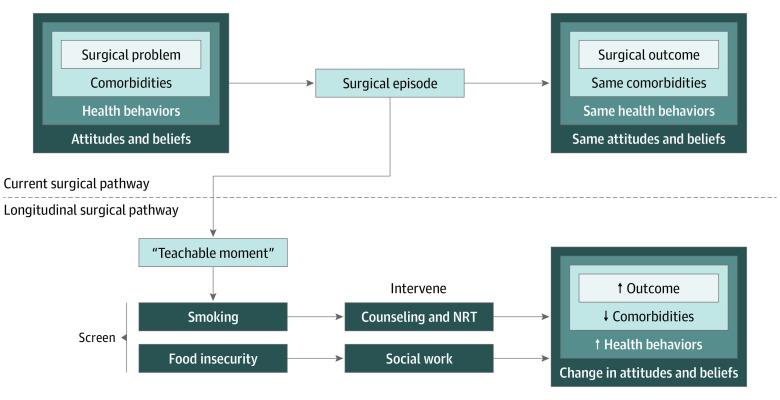
Conceptual Model of the Surgical Episode NRT indicates nicotine replacement therapy. The dashed line separates the current vs longitudinal surgical pathway. The upward arrows indicate “improved” or “better,” and the downward arrow indicates “fewer” or “decreased.”

The design of this program was informed by the conceptual model presented in Figure 1. Surgical care is a common touchpoint between patients and the health care system, with more than 50 million surgical procedures performed each year.^20^ Despite this fact, patients usually arrive at the end of their surgical journey without any health behaviors or unmet social needs having been addressed. The goal of this project was to evaluate the feasibility, effectiveness, and patient perceptions of efforts to address those needs within the pathway of surgical care. This study was deemed exempt by the University of Michigan institutional review board because this work was considered quality improvement and not research. Consent was obtained verbally before the start of the study. This report followed the Standards for Quality Improvement Reporting Excellence (SQUIRE) 2.0 reporting guideline for quality improvement studies.

We designed and implemented a program to screen patients for active tobacco use and food insecurity at their preoperative appointment and refer them to specific services to address each domain. This study was conducted between February 8 and August 31, 2021, at the University of Michigan preoperative clinic. This clinic performs preoperative evaluation of patients undergoing elective surgery at our institution and was chosen as the setting for this project because it serves as a centralized gateway for surgical patients. This clinic evaluates more than 2000 patients per month who are scheduled to undergo general, vascular, plastic, urologic, or gynecologic surgical procedures.^21^ More than 50% of all patients undergoing surgical procedures performed at our institution are evaluated in the preoperative clinic, and no specific procedures are considered ineligible for referral. Referral to this clinic is placed largely at the discretion of the surgeon or by the standard operating procedures of their department. All patients who were screened in the preoperative clinic and included in this study underwent their surgical procedure.

### Screening and Referral Intervention

The screening intervention consisted of 6 yes or no questions (Table 1). Screening for tobacco use consisted of a 3-question prompt asking (1) if the patient actively used tobacco, (2) if the patient was interested in resources to help quit smoking, and (3) if they wanted a referral to an outpatient counseling service (Tobacco Consultation Service [TCS]). Food insecurity screening consisted of 2 questions from the Hunger Vital Sign, a validated tool used to identify households at risk for food insecurity, as well as a third question asking if the patient wanted referral to our institution’s nonmedical needs assistance program.^22,23^ Implementation of this screening tool was guided by the advanced practice providers (APPs; physician assistants) who staff the clinic. This approach uses the local expertise of those who perform the daily work and ensures a pragmatic and sustainable approach. These 6 questions were incorporated into an interactive template in each patient’s history and physical examination in the electronic health record. Testing of the screening and referral intervention demonstrated that it added between 60 and 120 seconds to the preoperative appointment.

**Table 1.** Screening and Referral Tool

Tool	Answer
Tobacco use screening	
Active tobacco use?	Yes or no
Is patient interested in resources to help quit smoking?	Yes, no, or not applicable
Referral to Tobacco Consultation Service if yes?	Yes, no, or not applicable
Food insecurity screening	
Within the past 12 mo, did you worry that your food would run out before you got to buy more?	Yes or no
Within the past 12 mo, did the food you bought not last and you did not have money to get more?	Yes or no
Referral to Guest Assistance Program if yes?	Yes, no, or not applicable

If a patient screened positive for active tobacco use and agreed to a referral, the APP placed a referral to our institution’s TCS.^24^ This service involves multiple motivational interviewing sessions with licensed tobacco treatment specialists and provides recommendations for pharmacologic interventions, such as nicotine replacement. If a patient screened positive for food insecurity and agreed to a referral, the APP placed a referral to our institution’s Guest Assistance Program (GAP).^25^ This service involves a full nonmedical needs assessment by licensed social workers and connects patients with food assistance, transportation assistance, and financial assistance, among other services. Food assistance included a holistic assessment of patients’ challenges with access to quality nutrition in the context of their other nonmedical needs. Assistance included connection and referral to community programs, such as food pantries and meal delivery services. Referral to TCS and/or GAP was documented in the patient’s preoperative history and physical examination associated with that visit. All patients seen in the preoperative clinic were eligible for screening. This study did not independently evaluate outcomes among patients with both tobacco use and food insecurity. In the year prior to initiation of this pilot program, the baseline referral rate was 8.0% to TCS and 0% to GAP.

### Patient Surveys

Patients who received a referral to the TCS or GAP were contacted via telephone to complete follow-up surveys 30 to 90 days after surgery and between July 1, 2021, and March 31, 2022. The survey evaluated the patients’ attitudes toward addressing health behaviors and social determinants of health as part of a surgical episode as well as changes in their smoking and food insecurity status. A maximum of 4 attempts, spaced 3 to 5 business days apart, were made to administer the survey to each qualifying patient. Patients were informed that participation in the survey was voluntary, and informed consent was obtained verbally.

### Statistical Analysis

Data collection and analysis for this study were conducted using Qualtrics, version 2020-2022 (Qualtrics) and Microsoft Excel, version 2206 (build 15330.20196) (Microsoft Corp). Descriptive statistics were used to summarize the survey data.

## Results

**Figure 2.**
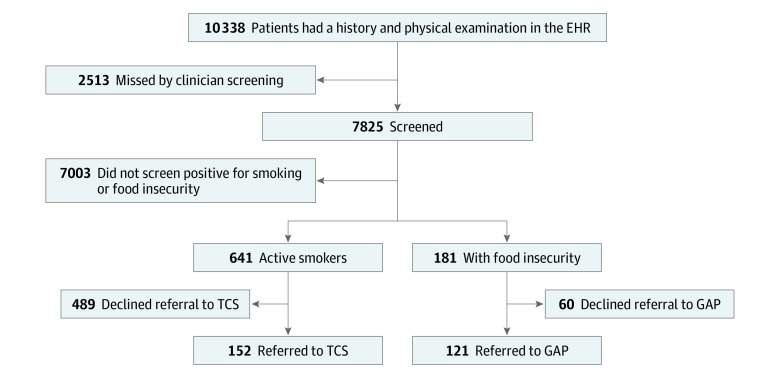
Summary of Patient Data Data shown are from February 8 to August 31, 2021. EHR indicates electronic health record; GAP, Guest Assistance Program; and TCS, Tobacco Consultation Service.

A total of 10 338 patients (6052 women [58.5%; 95% CI, 57.6%-59.5%]; 4286 men [41.5%; 95% CI, 40.5%-42.4%]; and mean [SD] age, 56.5 [17.9] years [95% CI, 56.2-56.8 years]) were evaluated in the preoperative clinic between February 8, 2021, and August 31, 2021 (Figure 2). Of 10 338 patients, 7825 (75.7%; 95% CI, 74.9%-76.5%) were screened for active tobacco use and food insecurity. Of the 7825 patients who were screened, 641 (8.2%; 95% CI, 7.6%-8.8%) screened positive for active tobacco use, and 181 (2.3%; 95% CI, 2.0%-2.7%) screened positive for food insecurity. Of the 641 active smokers, 152 (23.7%; 95% CI, 20.5%-27.2%) accepted a referral to the TCS. Of the 181 patients with food insecurity, 121 (66.9%; 95% CI, 59.5%-73.7%) accepted a referral to GAP.

Of the 152 patients who accepted a referral to the TCS, 78 (51.3%; 95% CI, 43.1%-59.5%) completed a telephone survey (Table 2). Of these 78 patients, 64 (82.1%; 95% CI, 71.7%-89.8%) agreed that the preoperative appointment was an appropriate time to discuss smoking cessation, and 46 (59.0%; 95% CI, 47.3%-70.0%) reported being more or much more motivated to quit smoking after engaging in this pilot program. Ultimately, at the time of the survey, 34 of 78 patients (43.6%; 95% CI, 32.4%-55.3%) reported quitting smoking since their preoperative visit, 18 (52.9%; 95% CI, 35.1%-70.2%) of whom reported quitting before their operation. Of the 41 patients who did not report quitting, 9 (22.0%; 95% CI, 10.6%-37.6%) had set a target quit date.

**Table 2.** Postreferral Survey of Patients Who Accepted a Referral to the Tobacco Consultation Service for Assistance In Smoking Cessation

Survey question	No. (%) (N = 78)
1. You were referred to Tobacco Consultation Services at your preoperative appointment. Do you feel like this was an appropriate time to discuss smoking cessation?	
Yes	64 (82.1)
No	5 (6.4)
Missing	9 (11.5)
2. On a scale of 1 to 5, how much did your surgery influence your thoughts to quit smoking, with 1 being much less motivated to quit and 5 being much more motivated to quit?	
1 = Much less motivated to quit	4 (5.1)
2 = Less motivated to quit	5 (6.4)
3 = No change in motivation to quit	21 (26.9)
4 = More motivated to quit	17 (21.8)
5 = Much more motivated to quit	29 (37.2)
Missing	2 (2.6)
3. Since your preoperative appointment, did you quit smoking cigarettes?	
Yes	34 (43.6)
No	41 (52.6)
Missing	3 (3.8)
4. If yes to question 3: do you remember if you quit before or after your surgery? (n = 34)	
Before	18 (52.9)
After	10 (29.4)
Do not remember	1 (2.9)
Other	5 (14.7)
5. If no to question 3: do you have a target quit date set? (n = 41)	
Yes	9 (22.0)
No	32 (78.0)

Of the 121 patients who accepted referral to GAP, 84 (69.4%; 95% CI, 60.4%-77.5%) completed a telephone survey (Table 3). Of these 84 patients, 69 (82.1%; 95% CI, 72.3%-89.7%) agreed that it was a good or very good idea for health systems to address nonmedical needs around the time of surgery. A total of 20 of 84 patients (23.8%; 95% CI, 15.2%-34.4%) were also connected with resources for nonfood-related needs, such as transportation, housing assistance, and financial assistance. Ultimately, 27 of 84 patients (32.1%; 95% CI, 22.4%-43.2%) reported no longer being insecure about food since their preoperative visit. Patients were considered no longer insecure about food if they answered no to both Hunger Vital Sign questions (ie, questions 2 and 3 in the survey).

**Table 3.** Postreferral Survey of Patients Who Accepted a Referral to the Guest Assistance Program for Food Insecurity

Survey question	No. (%) (N = 84)
1. Were you connected to any of the following resources? Select all that apply.	
Food or meal assistance program	18 (21.4)
Transportation service	10 (11.9)
Housing assistance	7 (8.3)
Financial assistance	11 (13.1)
Insurance assistance	10 (11.9)
Other	21 (25.0)
None	19 (22.6)
Missing	3 (3.6)
2. Since before your surgery (or since your referral to the Guest Assistance Program), have you been worried whether food would run out and you did not have the money to get more?	
Yes	47 (56.0)
No	35 (41.7)
Missing	2 (2.4)
3. Since before your surgery (or since your referral to the Guest Assistance Program), did the food you buy run out and you did not have the money to get more?	
Yes	40 (47.6)
No	42 (50.0)
Missing	2 (2.4)
4. Do you think your surgery was an appropriate time to address these needs?	
Yes	67 (79.8)
No	8 (9.5)
Missing	9 (10.7)
5. On a scale of 1 to 5, did you think the idea of addressing social needs (eg, food insecurity, housing insecurity, poverty, transportation needs) around the time of surgery was a good or bad idea?	
1 = Very bad idea	0
2 = Bad idea	1 (1.2)
3 = Neither good nor bad idea	10 (11.9)
4 = Good idea	21 (25.0)
5 = Very good idea	48 (57.1)
Missing	4 (4.8)

## Discussion

In this pilot project to address health behaviors and unmet social needs as part of a surgical episode, 3 major findings emerged. First, more than 75% of patients were successfully screened for smoking and food insecurity, demonstrating that a simple screening mechanism is feasible to implement into the workflow of a busy surgical clinic. Second, nearly one-fourth of smokers and more than two-thirds of individuals with food insecurity were referred to services to intervene on those conditions. This represents a 3-fold increase in referrals for smoking cessation (from 8.0% to 23.7%) and an increase in referrals for nonmedical needs assistance from 0% to 66.9%. Finally, during follow-up, not only did most patients agree that the perioperative period was an appropriate time to address these issues, but many patients reported no longer smoking or being insecure about food. In summary, these results speak to the feasibility, acceptability, and effectiveness of capitalizing on the surgical episode as an important opportunity to address patients’ foundational health.

Using specialty care pathways—such as surgical and procedural care—to address and intervene on foundational health is a novel solution. Although this setting has not traditionally been used in this fashion, an abundance of evidence suggests that it may be an opportune time to engage patients. First, the experience of undergoing surgery has been associated with significant and sustained health behavior change. Surgery is a transformative life event, and studies suggest that this experience can empower patients to adopt changes in their health they had previously not considered or were unable to make.^26^ For example, we observed a 43.6% tobacco quit rate compared with the national mean quit rate of 8.5%.^27^ In addition, several existing programs have demonstrated success in screening for and intervening on chronic health conditions around the time of surgery. These large-scale preoperative pathways screen patients for chronic conditions, such as smoking, obesity, diabetes, and stress, and are intended to address modifiable risk factors before surgery.^28,29^ Our pilot project differs in that it is focused on establishing longitudinal engagement around the time of surgery, even if health behaviors or unmet needs are not necessarily modified prior to surgery. In this way, the current project is like the “Making Every Contact Count” program in the United Kingdom,^30^ which uses any interaction with the National Health Service—from a routine eye examination to a minor outpatient operation—to screen patients for unhealthy behaviors and connect them with longitudinal resources. Such a shift in culture is timely and necessary given the still-low rates of screening in the hospital and physician practice settings for tobacco use, food insecurity, housing instability, utility needs, transportation needs, and interpersonal violence.^31,32^ All of these initiatives, including the current project, have the potential to add value by not only screening for modifiable surgical risk factors but by modifying health outcomes long after a patient’s surgical episode has ended.^33^

This pilot project also demonstrates how small, simple changes can be associated with measurable outcomes within complex health systems. Prior work^34,35^ has shown that quality improvement initiatives often fail or yield only short-term outcomes if they disrupt workflows or do not engage key stakeholders. A critical feature of the current project was that key stakeholders (namely, APPs) were identified and engaged from the beginning, and it was the APPs who guided the design and implementation of the screening tool. In this way, this pilot project used the principles of bottom-up change, respecting the expertise of those doing the work, and eliciting buy-in rather than mandating new procedures, which are central to a number of proven quality improvement methods and shown to increase the success and sustainability of these initiatives.^34,35^ Even with this pragmatic approach, nearly 25% of patients were not screened in the clinic, either owing to time constraint or process error. This figure represents a mean screening rate over the life of the program, and the screening rate improved to 90.0% of all patients (1515 of 1683) by the final month of our study. Simultaneously, this project used preexisting institutional resources. Rather than create new systems to intervene on tobacco use and unmet social needs, we coupled departments that provided those services with the preoperative evaluation, a connection that had not previously existed in this way. This approach of intentionally integrating these services into the surgical pathway may be particularly important for smoking cessation services, which have been shown to be underused despite being more available than ever.^36^ We believe that programs and outcomes similar to those described in this study can still be achieved even in institutions that do not have preexisting food insecurity and smoking cessation intervention programs. For example, patients could be referred to community resources, such as the Centers for Disease Control and Prevention’s national network of tobacco cessation quitlines, which are available in all 50 states. In short, this pilot project, which ultimately reached more than 10 000 patients, involved no new hires, no new technology, no additional resources, and no additional expense. The pragmatic nature of this pilot project suggests that other institutions could begin conducting similar projects at minimal effort today.

Although the initial results of this pilot project are promising, there are several ways in which it can be further improved. Efforts are needed to address the needs of the more than 75% of smokers who did not accept a referral to outpatient counseling. Screening at a preoperative visit implies that an operation has already been scheduled, and therefore the importance of smoking cessation might be underemphasized and the value of surgery as a teachable moment may be diminished. Consideration should be given to upstream interventions that are initiated by the surgeon, which may be particularly effective because patients expect their physician to discuss smoking around the time of an operation.^36^ Surgeons can help lead these changes by committing to addressing population health to fully capitalize on opportunities that provide higher-value surgical care regardless of whether or not those efforts are associated with immediate surgical outcomes. Second, survey response rates among both patient populations were low and may predispose our results to nonresponse bias. Although the low survey response rates may reflect the fact that individuals in the US are increasingly hesitant to answer telephone calls from unknown numbers, it also highlights the lack of longitudinal infrastructure to more easily capture long-term outcomes and ensure continuity of care.^37^ Future efforts should include multimodal survey contact methods (email, text message, and telephone), and survey collection should be structured and examined to ensure consistent results across modalities. The long-term sustainability and outcome of this initiative will be dependent on payment models that prioritize population health.^16^ Novel strategies, such as value-based reimbursement and billing modifiers, may be particularly useful and have previously been shown to affect practice change.^38^

### Limitations

This project has important limitations to consider. First, the cohort of patients who received the survey consisted only of patients who accepted a referral to smoking cessation or nonmedical needs services. It is possible that patients who accepted a referral were more likely to agree with the appropriateness and utility of this intervention. In addition, although we can infer patients’ level of engagement with the services to which they were referred through survey data, we were unable to collect process data explicitly measuring engagement beyond contact with the referred service. In addition, we did not capture important demographic or health-related patient characteristics that may influence engagement and survey responses. For example, the observed results may differ based on race, ethnicity, socioeconomic status, and comorbid conditions, such as lung disease, all of which have been shown to be associated with tobacco use and cessation. Moreover, a patient’s preexisting motivation to quit smoking may confound their willingness to accept a referral. Future work that seeks to understand clinical and patient factors associated with referral acceptance, barriers to accepting a referral, and the experience of those who decline a referral will be essential to increase the acceptability of interventions such as this. Second, the follow-up survey relied on self-reported tobacco cessation, which has been shown to be less reliable than other measures, such as urine cotinine tests. Despite this limitation, many studies that evaluate tobacco cessation rely on self-reported quit rates, and, moreover, even if the self-reported quit rate in this study were false in more than half of the cases (which is much higher than estimated), the observed quit rate would still have been significantly higher than the mean national quit rate.^39^ Furthermore, owing to logistical constraint, we did not concurrently assess quit rates for patients who did not receive this intervention, which is important to better understanding the effectiveness of this program. Third, the relatively low rate of tobacco use and food insecurity may reflect the unique population of patients undergoing elective surgery at a large academic center. Other studies of patients undergoing emergency surgery or receiving care in underresourced settings have demonstrated markedly higher rates of unaddressed chronic health behaviors and unmet social needs.^40^ Therefore, this type of project may be even more appropriate and more effective if deployed in those settings. Fourth, the long-term outcome of this intervention remains unclear in the present study. Tobacco recidivism, for example, can occur months to years after cessation, although similar work has shown a relatively close concordance between 30-day and 1-year quit rates after surgery.^41^ Future work that incorporates long-term outcomes and clinician attitudes about the program will be essential to scaling this intervention across complex health care systems.

## Conclusions

In this pilot project to address health behaviors and unmet social needs as part of a surgical episode, almost one-fourth of smokers were referred to smoking cessation counseling and two-thirds of patients with food insecurity were referred for nonmedical needs assistance. At follow-up, almost half of the patients referred to smoking cessation counseling reported quitting smoking, and almost one-third of patients referred for nonmedical needs assistance reported no longer being insecure about food. These results suggest that a simple, pragmatic intervention to address population health issues not traditionally addressed within specialty care pathways is both feasible to implement and effective.
